# Type 1 Diabetes Mellitus and Bariatric Surgery: A Systematic Review and Meta-Analysis

**DOI:** 10.1007/s11695-015-1999-6

**Published:** 2015-12-22

**Authors:** Hutan Ashrafian, Leanne Harling, Tania Toma, Christina Athanasiou, Nikolaos Nikiteas, Evangelos Efthimiou, Ara Darzi, Thanos Athanasiou

**Affiliations:** 1 Department of Surgery and Cancer, Imperial College London, 10th Floor, Queen Elizabeth the Queen Mother (QEQM) Building, Imperial College Healthcare NHS Trust at St Mary’s Hospital, Praed Street, London, W2 1NY UK; 2 Department of Bariatric Surgery, Chelsea and Westminster Hospital, London, UK; 3 Department of Hepato-pancreato-biliary (HPB) Surgery, Hammersmith Hospital, London, UK; 4 Department of Surgery, Athens University Medical School, Athens, Greece

**Keywords:** Type 1 diabetes mellitus, Type 2 diabetes mellitus, Diabetes, Bariatric surgery, Metabolic surgery, Body mass index, Intervention, Surgery, Weight loss, HbA1c, Glycosylated haemoglobin

## Abstract

**Background:**

Type 1 diabetes mellitus (T1DM) has a rising global prevalence. Although it is vastly outnumbered by type 2 diabetes mellitus rates, it remains a persistent worldwide source of morbidity and mortality. Increasingly, its sufferers are afflicted by obesity and its complications. The objective of the study is to quantify the effects of bariatric surgery on T1DM by appraising the primary outcomes of glycosylated haemoglobin (HbA1c), insulin requirements and body mass index (BMI). Secondary outcomes included blood pressure, triglycerides and cholesterol biochemistry.

**Methods:**

A systematic review of studies reporting pre-operative and post-operative outcomes in T1DM patients undergoing bariatric surgery was done. Data were meta-analysed using random effects modelling. Subgroup analysis and quality scoring were assessed.

**Results:**

Bariatric surgery in obese T1DM patients is associated with a significant reduction in insulin requirement (−48.95 units, 95 % CI of −56.27, −41.62), insulin requirement per kilogramme (−0.391, 95 % CI of −0.51, −0.27), HbA1c (−0.933, 95 % CI of −1.604, −0.262) and BMI (−11.04 kg/m^2^, 95 % CI of −13.49, −8.59). Surgery is also associated with a statistically significant reduction in systolic and diastolic blood pressure and a significant beneficial rise in HDL. Heterogeneity in these results was high, and study quality was low overall.

**Conclusions:**

Bariatric surgery in obese T1DM patients is associated with a significant improvement in insulin requirement and a significant though modest effect on HbA1c. These early results require further substantiation with future studies focusing on higher levels of evidence. This may offer a deeper understanding of diabetogenesis and can contribute to better selection and stratification of diabetic patients for metabolic surgery and future metabolic treatment strategies.

## Introduction

The global escalation of diabetes mellitus has been predominantly fuelled by the increase in type 2 diabetes mellitus (T2DM) that is associated with the concomitant rise in worldwide obesity and the metabolic syndrome. As a consequence, the proportion of worldwide diabetes patients suffering from T2DM has increased to approximately 90 % and those of type 1 diabetes mellitus (T1DM) has dropped to approximately 10 % of worldwide cases [1]. This however masks the finding that the prevalence of T1DM continues to rise by approximately 3 % annually, accounting for approximately 80,000 new cases worldwide every year [1].

T1DM typically presents in childhood and carries both genetic and autoimmune components [2, 3] that still necessitate treatment with insulin therapy (almost 90 years after the Nobel Prize for its discovery in 1923). It is associated with early mortality and the chronic morbidity of systemic vasculopathy, nephropathy and neuropathy in conjunction to the lifestyle burden of life-long insulin requirements. Both T1DM and T2DM share commonalities [2–4] that include disordered glucose metabolism and metabolic pathology that affects insulin secretion or utilization. They also share multi-systemic symptoms, although macrovascular complications are more common in T2DM whereas microvascular complications predominate in T1DM. Whilst T2DM is more strongly associated with obesity and the metabolic syndrome that results in an elevated systemic insulin resistance, there is an increasing recent trend where T1DM patients are also demonstrating increased adiposity and weight gain.

Bariatric surgery represents the most successful strategy in managing T2DM when associated with morbid obesity [5–7]. Its dramatic ‘bionic’ [6] multimodal effects include those on obesity reduction through weight loss in addition to the effects of enhancing systemic metabolism, decreasing insulin resistance and the metabolic syndrome. Despite the increased application and awareness of the role of bariatric surgery in the context of T2DM, the effects of metabolic surgical strategies on T1DM remain much less understood.

Our aim was therefore to systematically appraise the literature to identify the effects of bariatric surgery on patients with specifically T1DM.

## Methods

### Literature Search

**Fig. 1:**
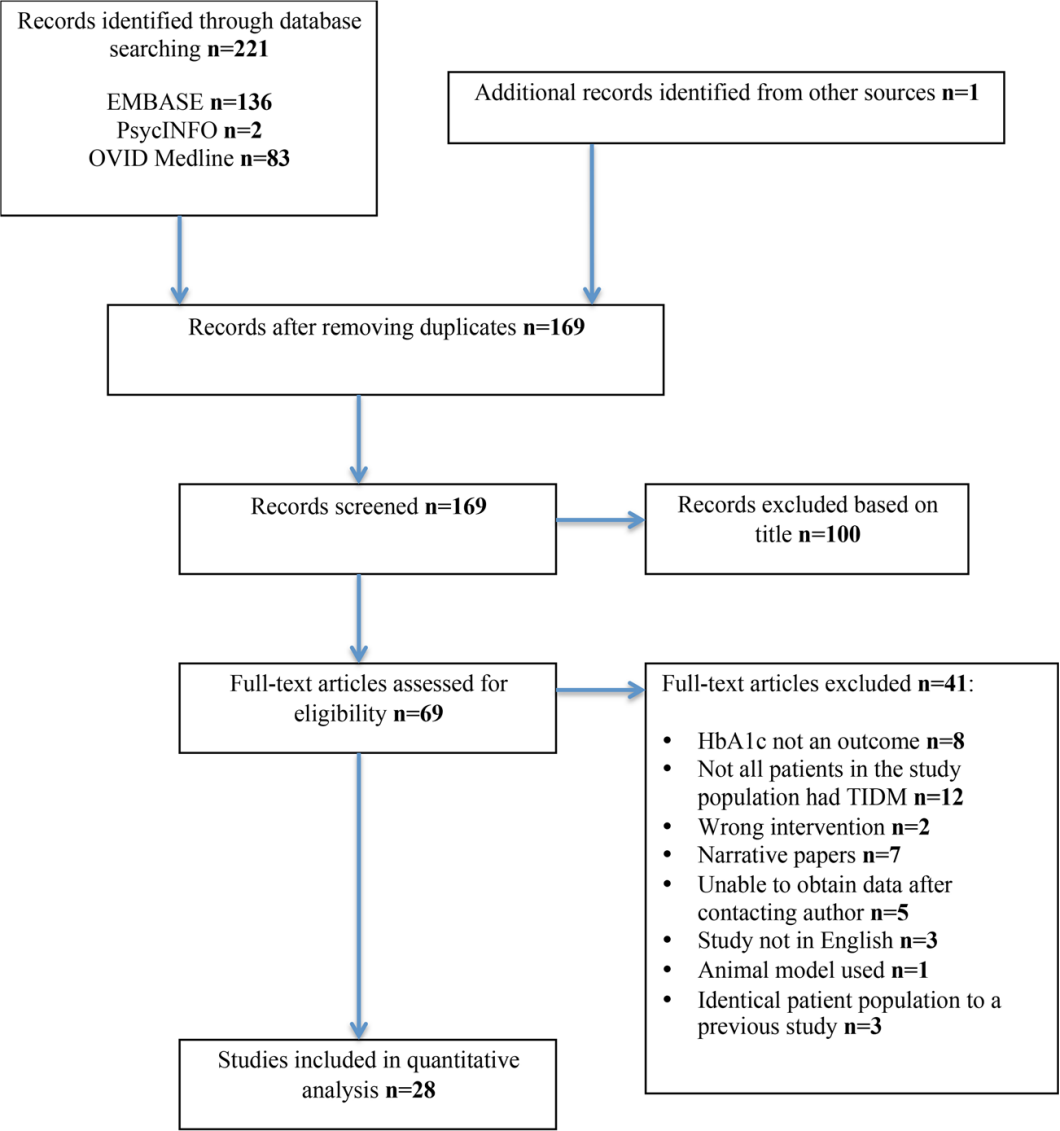
Search strategy

A literature search was performed using PubMed, Embase, Ovid and Cochrane databases using combinations of the terms ‘bariatric surgery’ or ‘metabolic surgery’ or ‘weight loss surgery’ and ‘Type 1 Diabetes’ or ‘Type 1’ and ‘Diabetes’. The last date for this search was 1 August 2015. Figure 1 outlines our search strategy. All studies are listed in Table 1.

**Table 1:** Bariatric surgical studies reporting on changes in type 1 diabetes mellitus

Author	Publication type	Study type	Study design	Subject number	Metabolic operation	Quality score
Czupryniak et al. 2004 [8]	Peer-reviewed journal	Case series	Retrospective	2	RYGB	4
Bastos et al. 2009 [9]	Conference abstract	Case report	Retrospective	1	RYGB	3
Muralidhara et al. 2010 [10]	Conference abstract	Case series	Retrospective	5	NS	3
Gagne et al. 2010 [11]	Conference abstract	Case series	Retrospective	5	RYGB	3
Mlawa et al. 2010 [12]	Conference abstract	Case report	Retrospective	1	RYBG	4
Spector et al. 2010 [13]	Conference abstract	Case series	Retrospective	7	RYBG, LAGB	4
Czupryniak et al. 2010 [14]	Peer-reviewed journal	Case series	Retrospective	3	RYGB	5
Mendez et al. 2010 [15]	Peer-reviewed journal	Case series	Retrospective	3	RYGB	5
Alvarez et al. 2011 [16]	Conference abstract	Case series	Retrospective	3	RYGB	4
Feilitzsch et al. 2011 [17]	Conference abstract	Case report	Retrospective	1	SG	3
Dorman et al. 2012 [18]	Conference abstract	Case series	Retrospective	6	RYGB, LAGB, DS	4
Silvestre Teruel et al. 2012 [19]	Conference abstract	Case series	Retrospective	2	NS	4
Carrasco et al. 2013 [20]	Conference abstract	Case series	Retrospective	7	RYGB	4
Esteves et al. 2013 [21]	Conference abstract	Case report	Retrospective	1	RYGB	3
Garciacaballero et al. 2013 [22]	Peer-reviewed journal	Case series	Retrospective	5	BAGUA	5
Chuang et al. 2013 [23]	Peer-reviewed journal	Case series	Retrospective	2	RYGB, VSG	4
Fuertes-Zamorano et al. 2013 [24]	Peer-reviewed journal	Case series	Retrospective	2	SADI-S	4
Raab et al. 2013 [25]	Peer-reviewed journal	Case series	Retrospective	6	RYGB, SG, BPD-DS	5
Dirksen et al. 2013 [26]	Peer-reviewed journal	Case report	Retrospective	1	RYGB	4
Reyes-Garcia et al. 2013 [27]	Peer-reviewed journal	Case report	Retrospective	1	RYGB	3
Stier et al. 2014 [28]	Conference abstract	Case series	Retrospective	10	RYGB, SG, BPD-DS	4
Blanco et al. 2014 [29]	Conference abstract	Case series	Retrospective	7	RYGB	4
Middelbeek et al. 2014 [30]	Peer-reviewed journal	Case series	Retrospective	9	RYGB	5
Tang et al. 2014 [31]	Peer-reviewed journal	Case series	Retrospective	6	RYGB, LAGB, SG	5
Brethauer et al. 2014 [32]	Peer-reviewed journal	Case series	Retrospective	10	RYGB, LAGB, SG	5
Lannoo et al. 2014 [33]	Peer-reviewed journal	Case series	Retrospective	22	RYGB, SG	5
Robert et al. 2015 [34]	Peer-reviewed journal	Case series	Retrospective	10	BPD, SG	5
Maraka et al. 2015 [35]	Peer-reviewed journal	Case series	Retrospective	10	BPD, SG	5

*RYGB* Roux-en-Y gastric bypass, *VBG* vertical sleeve gastroplasty, *BPD* biliopancreatic diversion, *DS* duodenal switch, *BPD*-*DS* biliopancreatic diversion with duodenal switch, *SG* sleeve gastrectomy, *SADI*-*S* single-anastomosis duodeno-ileal bypass with sleeve gastrectomy, *LAGB* laparoscopic adjustable gastric banding, *BAGUA* gastric bypass of single anastomosis, *NS* not specified

### Inclusion and Exclusion Criteria

All studies reporting pre-operative and post-operative liver biochemistry or liver histology (or both) were included. Studies were excluded for data inconsistency or overlapping data from other studies (for example, two studies used data from the same patients [8, 14]).

Meta-analysis was performed in line with recommendations form the Cochrane Collaboration and in accordance with Preferred Reporting Items for Systematic Reviews and Meta-Analyses (PRISMA) and Meta-analysis of Observational Studies in Epidemiology (MOOSE) guidelines [36]. Analyses were performed using Stata version 12 (StataCorp LP, College Station, TX).

Continuous data were investigated using weighted mean difference (WMD) as the summary statistic and proportion difference between histological outcomes were calculated and pooled through DerSimonian and Laird random effects modelling. Where not provided directly, standard deviations were imputed from available data. Quality assessment of each study was performed using the Newcastle-Ottawa scale.

## Results

Twenty-seven studies (Table 1) fulfilled the inclusion criteria, producing a pooled data set of 142 patients excluding repeated cohorts. All studies were non-randomized retrospective studies. The mean pre-operative BMI was 42.5, with a weight of 118.4 kg, and an insulin requirement of 108 units, or 0.86 U/kg. The mean diabetes duration was 20.8 years, and the mean age at operation was 39, with a follow-up time of 29.4 months (all weighted values). Ten studies were peer-reviewed case series (after excluding the lowest quality studies: abstracts and single cases); producing a pooled data set of 84 patients (these results were primarily used for discussion). For this subgroup, the mean pre-operative BMI was 42.06, the mean weight was 116.7 kg, and insulin requirement was 100.45 units, or 0.905 U/kg. The diabetes duration was 21.1 years, with a mean age at operation of 40.8 and a follow-up time was 31.8 months (all weighted values).

### Primary Outcomes

#### HbA1c

Pooled analysis of 22 studies demonstrated the weighted mean decrease in HbA1c was 0.788 % (95 % CI 0.334–1.24, *p* = 0.001, *I*^2^ = 80.7 %). Excluding the lowest-quality studies revealed 10 studies with an HbA1c decrease of 0.933 % (95 % CI 0.262–1.604, *p* = 0.006, *I*^2^ = 80.1 %).

#### Insulin Requirement

Pooled analysis of 17 studies demonstrated the weighted mean decrease in insulin requirement was 44.5 units (95 % CI 34.62–54.42, *p* < 0.00001, *I*^2^ = 51.2 %). Excluding the lowest-quality studies revealed 7 studies with an insulin requirement decrease of 48.95 (95 % CI 41.62–56.27, *p* < 0.00001, *I*^2^ = 0 %).

#### Insulin Requirement per Kilogramme

Pooled analysis of 15 studies demonstrated the weighted mean decrease in insulin requirements was 0.307 units (95 % CI 0.172–0.443, *p* < 0.00001, *I*^2^ = 77.1 %). Excluding the lowest-quality studies revealed 5 studies with an insulin requirement decrease of 0.391 (95 % CI 0.27–0.51, *p* < 0.00001, *I*^2^ = 0 %).

#### BMI

Pooled analysis of 19 studies demonstrated the weighted mean decrease in BMI was 12.917 kg/m^2^ (95 % CI 9.06–16.77, *p* < 0.00001, *I*^2^ = 93.7 %). Excluding the lowest-quality studies revealed 10 studies with a BMI decrease of 11.04 kg/m^2^ (95 % CI 8.59–13.49, *p* < 0.00001, *I*^2^ = 60.7 %).

### Secondary Outcomes

#### Systolic Blood Pressure

Pooled analysis of 2 studies demonstrated the weighted mean decrease in systolic blood pressure was 10.1 mmHg (95 % CI 1.05–19.16, *p* = 0.029, I^2^ = 0).

#### Diastolic Blood Pressure

Pooled analysis of 2 studies demonstrated the weighted mean decrease in diastolic blood pressure was 6.193 mmHg (95 % CI 0.78–11.61, *p* = 0.025, *I*^2^ = 0).

#### Triglycerides

Pooled analysis of 3 studies demonstrated the weighted mean decrease in TG was 25.124 mg/dL (95 % CI 9.265–40.98, *p* = 0.002, *I*^2^ = 12.4 %). Excluding the lowest quality study revealed 2 studies with a TG decrease of 11.04 mg/dL (95 % CI 8.59–13.49, *p* < 0.00001, *I*^2^ = 60.7 %).

#### LDL

Pooled analysis of 3 studies demonstrated the weighted mean decrease in LDL was 19.010 mg/dL (95 % CI 8.87–46.89, *p* = 0.181, *I*^2^ = 25.9 %). Excluding the lowest quality study revealed 2 studies with a LDL decrease of 9.54 mg/dL (95 % CI 8.8–27.9, *p* = 0.3, *I*^2^ = 0 %).

#### HDL

Pooled analysis of 3 studies demonstrated the weighted mean increase in HDL was 14.07 mg/dL (95 % CI 5.389–22.75, *p* = 0.001, *I*^2^ = 0 %). Excluding the lowest quality study revealed 2 studies with a HDL increase of 13.51 mg/dL (95 % CI 3.25–23.77, *p* = 0.01, *I*^2^ = 0 %).

#### Weight Change (kg)

Pooled analysis of 6 studies demonstrated the weighted mean decrease in body weight was 37 kg (95 % CI 23.9–50.0, *p* < 0.00001, *I*^2^ = 62.2 %). Excluding the lowest quality studies revealed 4 studies with a weight decrease of 27.99 kg (95 % CI 19.29–36.69, *p* < 0.00001, *I*^2^ = 0 %).

## Discussion

Overall, our analysis demonstrates that bariatric surgery for severely obese type 1 diabetes mellitus (T1DM) patients offers a significant reduction in biochemical markers of diabetes status and insulin requirements. Insulin doses dropped dramatically by approximately 50 % (both absolute values and those indexed to weight), and heterogeneity was minimal when excluding the lowest quality studies. Glycosylated haemoglobin (HbA1c) levels were also consistently decreased by a modest amount of 0.93. As expected, there was a significant reduction in post-operative body mass index. Note however must be taken that the literature reporting on bariatric surgical outcomes in T1DM patients remains in its early phases; the majority of papers in this field are of low evidence. Consequently, the results from these data require judicious caution to their inherent biases. Nonetheless, as we identified over 142 reported T1DM subjects undergoing bariatric procedures, we felt that appraisal of the current literature would offer some useful insights. The re-analysis of our results, when excluding the lowest-quality studies (case reports and abstracts), continued to reveal significance in beneficial outcomes and was even more pronounced for insulin requirements and HbA1c.

Our results also identified favourable outcomes for other diabetes confounders such as blood pressure, triglyceride and cholesterol (LDL and HDL), although these endpoints were reported in only a handful of studies. These all demonstrated significant decreases after surgery excluding HDL, which demonstrated a significant and advantageous increase. The results for triglycerides and LDL however did not retain significance when excluding abstracts and case reports. Whilst there is some evidence that particular bariatric procedures such as the Roux-en-Y gastric bypass offer superior anti-diabetic effects on T2DM in the short to midterm [7] compared to other procedures such as the sleeve gastrectomy, this currently remains debated and inconsistent for all outcomes. Based on the studies in this analysis, there was no robust comparative breakdown of cases and diabetic endpoints to clarify whether various bariatric procedure types offered differentiating effects on T1DM outcomes.

Although T1DM patients are considered higher-risk surgical candidates, the reported cases did not consistently specify significant trends or concerns in post-operative complications [32]. Occasional post-operative hypoglycaemia was described in one adult [14] and two adolescents [23], one of whom also suffered from diabetic ketoacidosis (DKA). Additionally, post-operative pneumonia occurred in one subject [8]. However, the majority of the studies were not necessarily designed to look for complications in these cohorts so that formal prospective studies in this area are necessary. We however feel that in view of their complex metabolic, endocrinological and multi-system pathologies, bariatric patients with T1DM should be managed and followed up with caution within the context of a multi-disciplinary healthcare team.

The dramatic effects of bariatric surgery on T2DM have revolutionized the management of these patients [5–7, 37] and have generated the contemporary concept of these operations as ‘metabolic surgery’ in view of their ‘bionic’ [6] anti-diabetic metabolic effects. This has been associated with a myriad of other beneficial metabolic actions that demonstrate activity on the systemic metabolism and specific organ systems including an improvement in cardiac disease [38], renal disease [39], non-alcoholic fatty liver disease [40], sleep apnoea [41] and even cancer [42]. The driving mechanisms associated with these actions comprise of the BRAVE effects [5, 43] (bile flow changes, restriction of stomach size, anatomical gastrointestinal rearrangement, vagal manipulation, enteric hormonal modulation) of bariatric surgery that in turn activate several systemic weight-independent and weight-dependent drivers of diabetes resolution. These include those of caloric restriction, gut microbiomic fluxes, adipokine profiles, decreased adipose-related inflammatory load and intestinal gluconeogenesis [5, 44].

One large systematic review of bariatric surgery after T2DM [37], comprising of 621 studies with 135,246 patients, revealed that for subjects with a mean age of 40.2 years and BMI of 47.9 kg/m^2^, bariatric surgery offered a decrease in BMI by 14.5 kg/m^2^, and within 2 years, there was a drop in HbA1c by 2.13 % and a drop in insulin dose by 97.98 units. As a cursory comparison, our results based on much fewer subjects suffering from T1DM in the peer-reviewed papers is a decrease in BMI by 11 kg/m^2^, a drop in HbA1c by 0.933 % and a drop in insulin dose by 48.95 units.

**Fig. 2:**
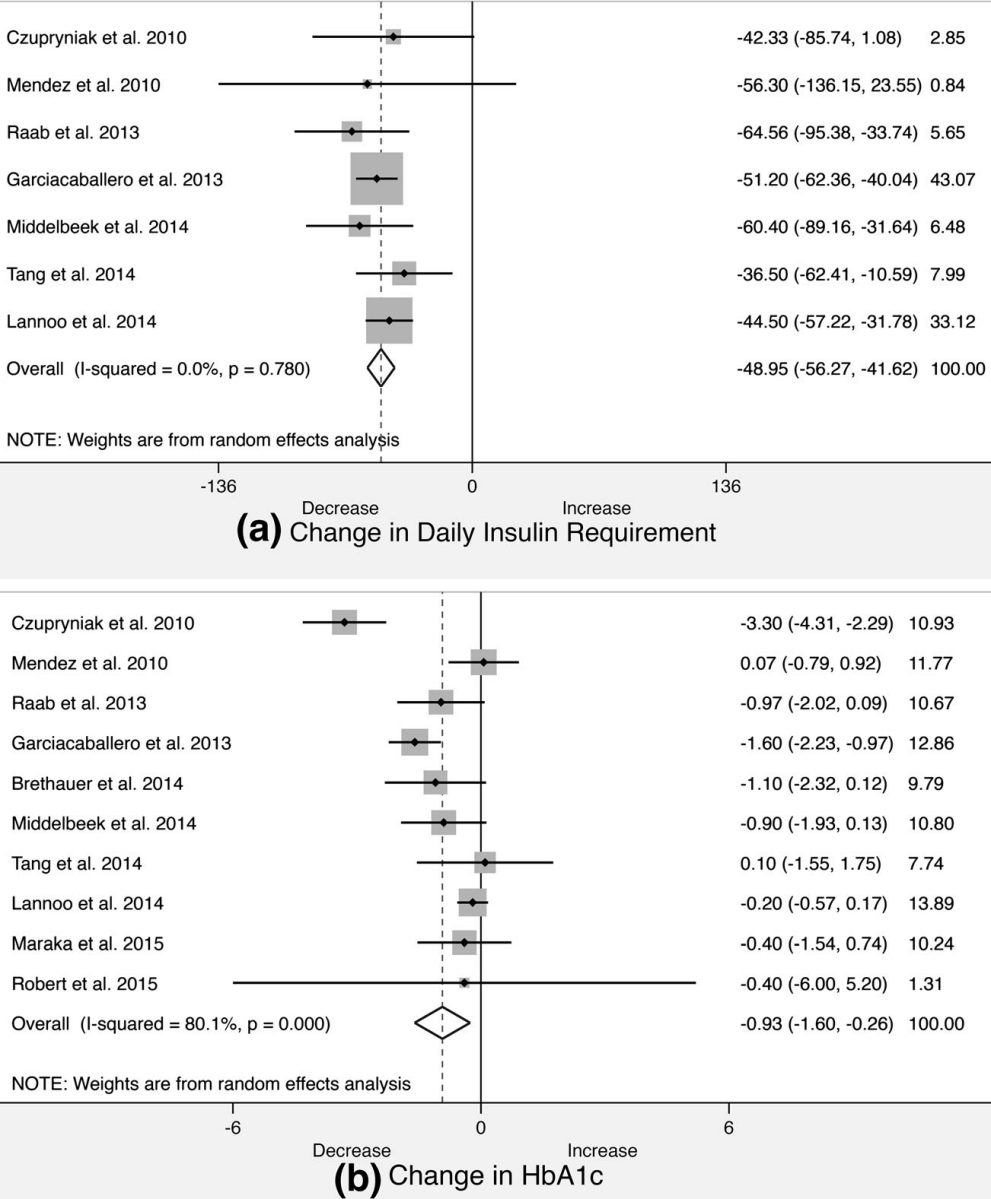
Forest plots demonstrating changes in **a** insulin requirement, **b** HbA1c following bariatric surgery in type 1 diabetes mellitus patients

**Fig. 3:**
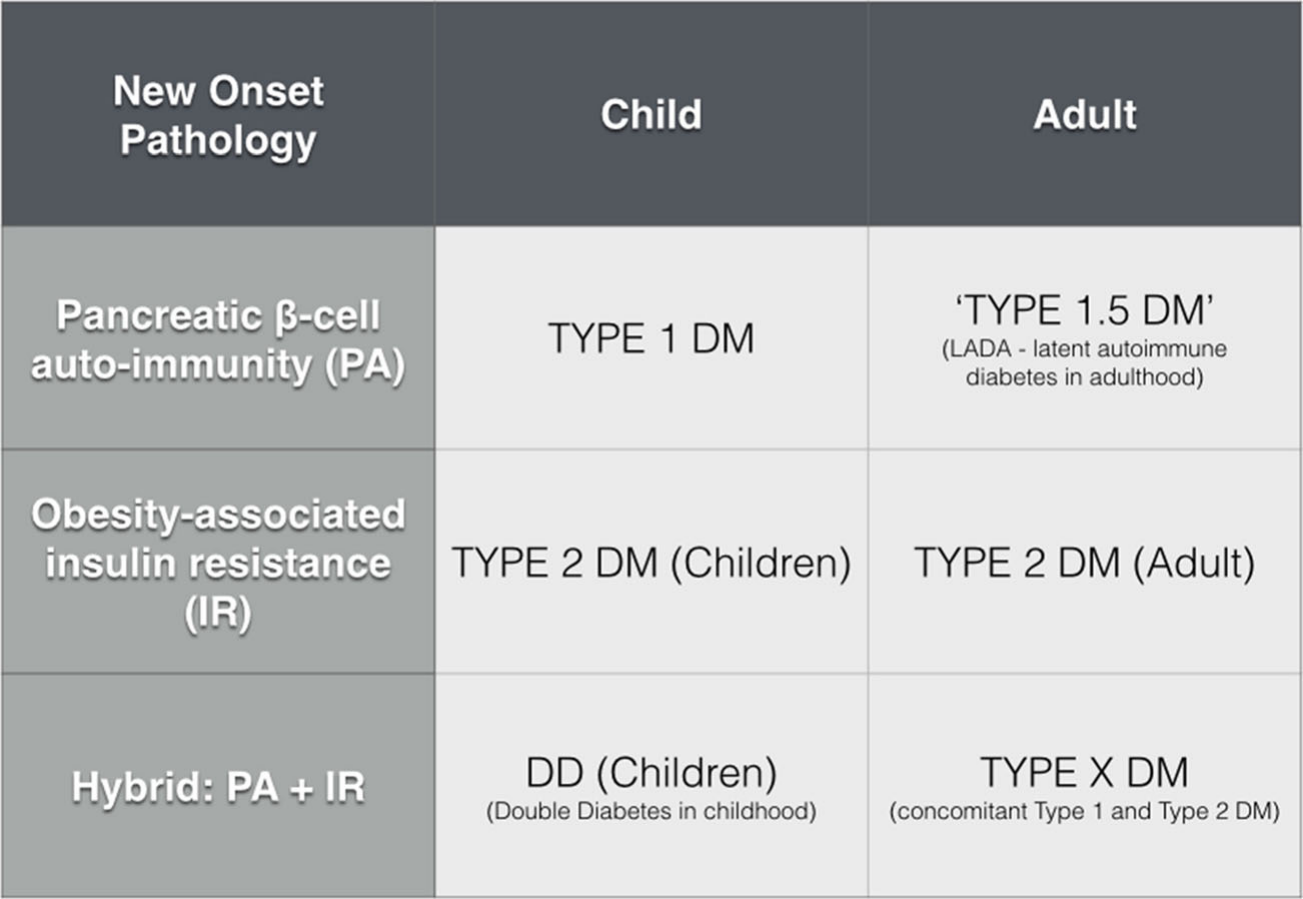
Spectrum of type of diabetes mellitus

The finding that bariatric surgery can offer significant decreases in both insulin requirements and HbA1c levels (even modestly) in T1DM contradicts the classical view of this disease as purely an autoimmune condition that irreversibly impairs pancreatic β-cell insulin secretion; for this would not account for any improvements in insulin secretion following surgery. It is however increasingly recognized that T1DM occupies a role within a spectrum of glucose metabolic pathology (Fig. 2). Diabetic conditions [45] on this spectrum include latent autoimmune diabetes in adulthood (LADA or type 1.5 diabetes) where pancreatic B cell auto-antibodies are identified de novo in subjects older than 35 or alternatively children who suffer from ‘double diabetes’ [46] because they are obese and insulin-resistant (and in T2DM) but also demonstrate an auto-antibody profile similar to T1DM (Fig. 3).

Based on the results from bariatric surgery in obese T1DM patients, we suggest that these patients suffer from an adult variant of ‘double diabetes’. Here, subjects demonstrate T1DM in infancy but subsequently acquire the factors of obesity and insulin resistance in keeping with T2DM later on in adolescence and adulthood. When these so-called type X diabetics undergo bariatric surgery, the metabolic surgical effects predominate in minimizing the T2DM components of disease, although the patients still suffer from inherent type 1-type β-cell dysfunction and therefore require baseline insulin therapy. Whilst this may offer a provisional framework for appraising the effects of bariatric surgery on T1DM patients, considerations still exist regarding the exact metabolic benefits of patients with pure T1DM. There are controversial reports of regenerative pancreatic β-cell hyperplasia and nesidioblastosis [5] after Roux-en-Y gastric bypass (particularly speculative for human subjects), the dramatic post-operative shifts leading to supra-physiological insulin release in the context of decreased insulin resistance are better recognized. Effects such as these may contribute to reducing β-cell dysfunctional load, although the precise mechanistic role of these operations in T1DM patients requires a cohesive research strategy.

In order to better select and clarify the effects of bariatric surgery on T1DM patients, there are several future steps that may be consequential from this analysis. There is a need for larger studies of better quality to achieve higher levels of research evidence regarding metabolic surgery for T1DM. Patients require their diabetes to be better characterized, and patients with the possibility of type X disease (Fig. 3) require more in-depth biochemical and clinical classification. Studies focusing on comparisons between specific patient diabetic cohorts and specific procedure types are encouraged and should take place in parallel with appropriate mechanistic studies clarifying the most appropriate procedure for each individual’s diabetic subgroup.

## Strengths and Limitations

This study offers the first quantifiable combined measure of the literature regarding bariatric surgery in T1DM patients. The literature in this field remains in its infancy, so that all studies currently occupy low evidence levels. Heterogeneity in the results represents a significant interpretive limitation. Furthermore, study design without matching, controls or prospective analysis in addition to small patient numbers, variability in procedures and variable follow-up time may lead to selection and reporting biases and therefore preclude definitive conclusions regarding outcomes.

## Conclusions

The effect of bariatric surgery on obese T1DM patients suggests that these procedures are associated with a significant decrease in insulin requirement and a significant though modest improvement in glycosylated haemoglobin (HbA1c). Metabolic operations also offer improvements in diabetic haemodynamic and biochemical confounders including HDL and systolic and diastolic blood pressure. Significant heterogeneity and lower levels of study evidence limit our interpretation of the results. The finding that there is a significant reduction in biomarkers of type 1 diabetes mellitus and accompanying insulin requirements arguably supersedes the traditional paradigm that these operations are only for type 2 diabetes mellitus as they cannot reverse the inherent pancreaopathy of T1DM. Whether these operations reduce the type 2 diabetic elements of insulin resistance associated with obesity in this patient group or whether there are additional T1DM targeted mechanisms requires further in-depth research. Further studies that include randomized controlled trials in conjunction with mechanistic studies are therefore necessary to better clarify the role of bariatric surgery and innovative metabolic interventions in the future management of the persistently pernicious type 1 diabetes mellitus.
